# A Multi-Modal Family Peer Support-Based Program to Improve Quality of Life among Pediatric Brain Tumor Patients: A Mixed-Methods Pilot Study

**DOI:** 10.3390/children7040035

**Published:** 2020-04-20

**Authors:** Justin G. Wilford, Ruth McCarty, Lilibeth Torno, Grace Mucci, Nadia Torres-Eaton, Violet Shen, William Loudon

**Affiliations:** 1 Department of Population Health & Disease Prevention, University of California Irvine, Irvine, CA 92697, USA; 2 MaxLove Project, Orange, CA 92868, USA; 3 Chinese Medicine and Acupuncture, CHOC Children’ s Main Campus Orange, Orange, CA 92868, USA; openmindmodalities@gmail.com; 4 Department of Oncology, CHOC Children’ s Main Campus Orange, Orange, CA 92868, USA; ltorno@choc.org (L.T.); vshen@choc.org (V.S.); 5 Department of Pediatric Psychology, CHOC Children’s Main Campus Orange, Orange, CA 92868, USA; gmucci@choc.org (G.M.); dr.torreseaton@gmail.com (N.T.-E.); 6 Department of Neurosurgery, CHOC Children’s Main Campus Orange, Orange, CA 92868, USA; Wgloudon@gmail.org

**Keywords:** childhood cancer, pediatric brain tumor, quality of life, peer support, acupuncture

## Abstract

Background: Pediatric brain tumor (PBT) survivors and their families are at risk for diminished psychosocial and quality of life outcomes. Community-based programs that leverage peer support in the context of integrative modalities such as traditional Chinese medicine (TCM) represent a promising avenue for meeting the multidimensional needs of survivors and their families. Methods: Parents and children were enrolled in a 12-week program that included weekly group TCM, a moderated private Facebook support group designed through social support and modeling theory, and weekly parent-only health behavior education and yoga. Process measures and quantitative and qualitative survey data was collected to gauge participant adherence, acceptability, and satisfaction, as well as exploratory outcomes. Results: Eleven parents completed surveys at all time points. Six of nine families attended at least 80% of the group TCM sessions, and eight of nine families interacted in the Facebook support group at least five days a week. Parents reported high levels of satisfaction and perceived benefits for the program. Baseline emotional distress, health behaviors, and QoL measurements improved during the three-month intervention. Qualitative data indicated parents perceived both in-person and the Facebook group peer support contributed to the benefits of the program. Conclusion: This feasibility study demonstrated that a multimodal peer support-based intervention that included in-person and online group interaction is feasible and acceptable to parents of pediatric brain tumor patients. Further research on interventions for caregivers that include in-person and online group-based peer support is warranted, with the goal of exploring similar outcomes in other childhood cancer diagnoses.

## 1. Introduction

Childhood cancer survivors are at significantly higher risk than age-matched peers for lifelong impaired quality of life (QoL) and general health [1,2]. Pediatric brain tumor patients are at even greater risk for long-term decrements in health and QoL [3,4,5]. Some of these risks may be mitigated through the early practice of preventive health behaviors such as physical activity, optimal sleep hygiene, healthy dietary patterns, and consistent positive social interactions [6,7,8,9,10,11,12]. However, clinical capacity to develop, implement, and evaluate programs that improve these practices has historically proven limited [13,14].

Community-based programs represent a promising avenue for health promotion program development among pediatric cancer survivors. Due to the consistent increase in survival rates attended by late effects and developmental delays, childhood cancer is experienced by many families as a chronic illness [15,16,17,18]. Supportive peer relationships for parents are particularly important in this context, as parents face late-effect management [19], uncertainty over their child’s future [20,21], and feelings of social isolation [22,23]. Not only is peer support associated with lower emotional distress and improved disease management [24,25,26], but it is also a potentially effective mechanism for promoting and maintaining preventive health behavior changes [27,28,29]. The development of peer support-based programs thus represents a promising avenue for improving a range of quality of life and lifestyle outcomes for parents and children affected by childhood cancer.

Since many pediatric brain tumor (PBT) patients and survivors experience decrements in psychosocial and physical QoL, traditional Chinese medicine (TCM) modalities represent a promising adjunct to peer support-based preventive health promotion. TCM modalities have been shown to significantly reduce pain [30,31,32], fatigue [33,34,35,36], and increase psychosocial and physical QoL [37,38,39]. Beneficial changes in these domains are necessary for families of PBT patients and survivors to engage in critical survivorship-related preventive health behaviors, such as diet, physical activity, optimal sleep hygiene, and consistent positive social interaction.

While TCM may improve aspects of QoL to allow for positive survivorship-related health behavior changes, additional support may be needed to begin and maintain new behaviors. Social support and peer support more specifically have shown promise in providing informational and emotional resources that motivate sustained behavior change. Peer support interventions have been studied in both face-to-face and online contexts but less often in combination. Combining the two modes of interaction may improve outcomes by leveraging the benefits of each modality. Face-to-face peer support mechanisms, such as active listening and affective identification [40], complement other peer support mechanisms, such as homophily [27] and social modeling [41], that require more consistent interactions more easily facilitated online. Social media, in particular, is a promising online platform for parent peer support, with over 70% of parents in the U.S. using social media, crossing all socioeconomic categories [42]. Furthermore, parents of patients and survivors are already connecting with each other on social media, building significant relationships and sharing health-related information [43,44].

For these reasons, an intervention that combines TCM with peer support-based preventive health promotion represents a promising solution for PBT families coping with diminished psychosocial and physical QoL. Despite the promise of combining these intervention components for families affected by childhood cancer, no studies to our knowledge have investigated the feasibility and acceptance of such an approach. The aim of this study was to investigate the acceptance and feasibility of a TCM and peer support-based health promotion intervention (The Ohana Project) to improve preventive health behaviors and quality of life among parents and children affected by pediatric brain tumors using a mixed-methods approach.

## 2. Materials and Methods

### 2.1. Recruitment and Participants

Participants were recruited as a convenience sample through information fliers presented to families at the Children’s Hospital of Orange County’s (CHOC Children’s) Neurosurgery Clinic in Orange, California, USA. Fliers described the pilot study as including traditional Chinese medicine (TCM), preventive health and QoL-related education, and online peer support. Parents were invited to contact study coordinators for further information. Eligibility criteria for parents were (1) 18 years and older; (2) English speaking; (3) access to web-enabled phone, tablet, or computer; (4) parent to a child diagnosed with a brain tumor at CHOC Children’s; (5) child was cleared by referring physician to receive acupuncture; (6) child was 6 months to 14 years old; and (7) child lived with at least one parent. Exclusion criteria were (1) child was on end-of-life care, (2) child was concurrently diagnosed with a life-threatening co-morbidity, and (3) parent or child was unable to participate due to travel restrictions. Exclusion criteria did not include time from diagnosis, because the preliminary investigation suggested that parents sought peer support primarily based on diagnosis. Parents did not find the stage of treatment to be an important factor in sharing peer support [19,22]. Language restrictions were due to the peer support group nature of the intervention, which required that all participants in a small group speak the same language. All subjects gave their informed consent for inclusion before they participated in the study. The study was conducted in accordance with the Declaration of Helsinki, and the protocol was approved by the Ethics Committee of the Children’s Hospital of Orange County (170105).

### 2.2. Intervention

The study’s protocol was approved by the Institutional Review Board of CHOC Children’s Hospital. The “Ohana Project” was designed by a multidisciplinary team that included a health behavior scientist who was also a parent of pediatric brain tumor patient (JW), a licensed pediatric TCM practitioner (RM), a pediatric neurosurgeon (WL), and a pediatric psychologist specializing in oncology (NT-E). All components of the study took place online and in a community-based space jointly administered by MaxLove Project, a U.S.-based non-profit, and Open Mind Modalities, a private TCM clinic. 

The theoretical frameworks that guided the design of the program were peer support theory and homophilous modeling. Peer support theory, as articulated by Dennis [40] and Thoits [45], predicts that individuals experiencing similar health crises provide each other with unique forms of support that may decrease emotional distress and improve self-care. Homophilous modeling, as articulated by Centola [27,46], predicts that individuals who know they share similar defining characteristics such as age, gender, and health conditions are more likely to change behaviors in a peer group context—even online—where at least one other peer is engaged in the targeted behavior. Thus, each component of the program was designed for consistent peer interaction. Targeted health behaviors included: diet with a focus on increasing unprocessed foods and limiting processed foods, promoting physical activity, developing healthy sleep habits, and developing parent stress management techniques.

**Figure 1:**
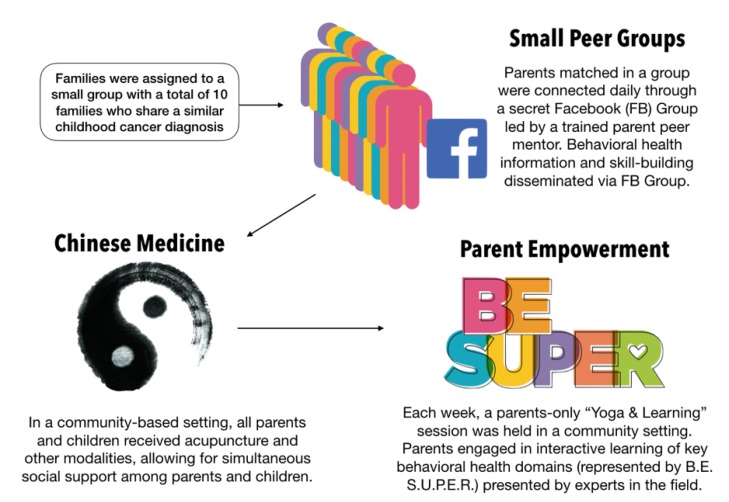
A visualization of the Ohana Project intervention structure.

Eligible parents received individual orientations that included the provision of program materials, assistance with Facebook registration, scheduling, and sharing contact information. The 12-week program consisted of three components (represented in Figure 1): (1) a moderated private Facebook group with asynchronous daily communication (i.e., communication in which the sender may not receive an immediate response, which allows for communication between those with diverse schedules who may communicate whenever it is convenient), (2) weekly group TCM for parents and their children (2 h/week) in a community setting, and (3) weekly parent-only health behavior education and yoga (2 h/week) in a family center jointly administered by MaxLove Project and a private TCM clinic. The Facebook group was a “secret” group, which meant that membership was by invitation only. In “private” Facebook groups, the group’s existence, individual membership, and posts cannot be seen by nonmembers. The group was led by the research coordinator (JW), trained in preventive health behavior change techniques, who was also a parent of a pediatric brain tumor patient and, thus, served as a peer mentor. The group was designed to activate homophilic-modeling behavior change by providing daily prompts and encouragement to engage in preventive health actions with their children, post photos and experiences in the group communicating these actions, and share comments on each other’s posts. 

Outpatient TCM was provided at the study site by licensed TCM practitioners in a group setting that allowed parents to engage in open social support by freely talking to one another while their children played with toys, interreacted with one another, or engaged in art. TCM modalities included acupuncture, acupressure, aromatherapy, cupping, and moxibustion. Acupuncture points were selected based on the patient’s presentation. Points were also selected for modality (i.e., acupuncture, acupressure, and moxibustion) based on the patient’s presentation.

Finally, the weekly parent-only health behavior education and yoga sessions were designed through active learning and group support principles. The health behavior education components focused on evidence-based health behaviors represented by the acronym B.E. S.U.P.E.R. Each letter stood for a health behavior, such as whole-food nutrition (E for Eat Super Fierce Foods), physical activity (U for Unleash Your Super Strength), sleep hygiene (S for Sleep Super Peacefully), and stress management (P for Practice Super Mindfulness), that has been shown to improve quality of life and reduce long-term health risks in both children and adults. Each educational session was designed and led by a clinician in the related field: a clinical dietitian for nutrition, a physical therapist for physical activity, a public health educator for sleep behaviors, and a psychologist for stress management. The yoga sessions were led by a hospital-based physical therapist. 

### 2.3. Measures

Process measures of intervention adherence, gathered throughout the intervention, included TCM appointment attendance; Facebook group participation (posting, commenting, and brief responses such as “like” or “love”); health behavior education; and yoga attendance. All other measures were collected through online surveys (REDCap) using weblinks given to parents one week prior to the intervention’s start (T1), the week following the intervention’s completion (T2), and 3 months post-intervention (T3). Parent and child demographics and child disease and treatment characteristics were collected at T1. Likert-type scale and open-ended acceptability/usability questions were given at T2. Parents were surveyed about the perceived level of convenience and value of the individual components (TCM, Facebook group, education, and yoga), as well as broader features of the intervention: length, time burden, helpfulness, impact on child, and parent well-being and recommendations offered to future parents. There were four open-ended questions, and they inquired about the overall perceived value (“What was the most valuable part of the Ohana Project for you and your child?”), satisfactory, and unsatisfactory components (“If there were one part of the Ohana Project that you could remove, what would it be?” and “If there were one part of the Ohana Project that you could add, what would it be?) and impressions they would share with other parents (“What would you tell another cancer family about the Ohana Project?”). “The Patient-Reported Outcome System” (PROMIS) [47] was used for all exploratory measures, which were given at all time points. These included anxiety (short form v.1.0, 4a), depression (short form v.1.0, 4a), sleep disturbance (short form v.1.0, 4a), emotional support (short form v.2.0, 4a), informational support (short form v.2.0, 4a), child physical activity (short form v.1.0 parent-proxy 4a), child cognitive function (short form v.1.0 parent-proxy 7a), and child QoL (global health 7, parent-proxy v.1.0).

### 2.4. Analysis

Participant demographics, disease and treatment characteristics, process measures, Likert-type acceptability/usability outcomes, and exploratory outcomes were descriptively analyzed using frequencies, means, and standard deviations with STATA 14.2 (College Station, TX, USA). Inferential statistics were not performed given the small sample size and the pilot nature of the study. Responses to open-ended questions were investigated using thematic analysis for representative themes by two researchers (JW and RM).

## 3. Results

Fifteen parents, representing 12 children, were screened for eligibility, with two found ineligible because of the children’s ages. Twelve parents representing 10 children were found eligible, provided informed consent, and completed the T1 survey and began the intervention. One parent and one child withdrew from the study after week three due to end-of-life disease progression. Eleven parents, representing nine families, completed surveys at all three time points. As shown in Table 1, the sample of enrolled parents and children was composed of seven Hispanic and five Caucasian parents with five Hispanic and five Caucasian children. The mean age of parents and children was 38.3 and 7.8, respectively. Six of the 10 children had received surgery, chemotherapy, and radiation. Five of the children were still in treatment, and five were off treatment.

### 3.1. Adherence

**Figure 2:**
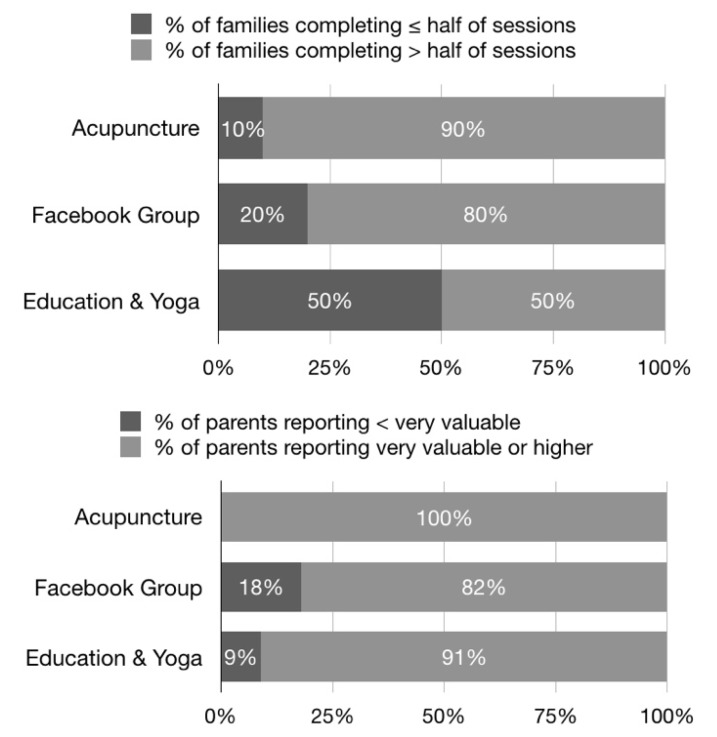
Summary of parent adherence and perception of values.

Figure 2 shows summaries of program adherence and satisfaction. Throughout the 12 weeks of the intervention, a minimum of six and a maximum of 10 families were present at the weekly group TCM sessions. Six of the nine families who completed the study attended at least 10 of the 12 weekly TCM sessions. A minimum of two and a maximum of eight families were present at each of the 12 weekly health behavior education and yoga sessions. Five of the nine families who completed the study attended at least seven of these sessions. Regular participation (five days of posting, commenting, or responding in a week) in the Facebook peer support group ranged from a maximum of 10 (week two) and a minimum of four (week seven) families. Eight of the nine families who completed the study averaged five days of engagement per week in the Facebook group. Among these nine families, parents recorded a total 3895 unique posts, comments, or responses in the Facebook group, ranging from 187 to 990 over the 12 weeks. 

### 3.2. Usability and Acceptability 

Facebook groups’ mean convenience score was 4.2 on a Likert-type scale of 1 to 5, with 6 of 11 parents reporting the highest category of “Very Convenient”. Facebook groups received a mean score for a perceived value of 4.6/5, with 9 of 11 responses of “Very” or “Extremely valuable”. TCM’s mean convenience score was 4.5/5, with 7 of 11 responses of “Very convenient”. TCM’s mean perceived value score was 4.9/5, with 11 of 11 responses of “Extremely” or “Very valuable”. Education and yoga sessions’ mean convenience score was 2.8/5, with three responses of “Convenient” and four responses of “Difficult” or “Very Difficult” to attend. Education sessions’ mean perceived value score was 4.6/5, with 11 of 11 responses of “Extremely” or “Very valuable”. Yoga received a mean score for a perceived value of 4.3/5, with 8 of 11 responses of “Extremely” or “Very valuable”. Ten of 11 parents reported the intervention to be “A little” or “Not at all” burdensome, and 10 of 11 reported it to be either “Too short” or “About the right length”.

Nine of 11 parents reported that the overall intervention made a “Great deal” or “A lot” of difference in their “child’s well-being”, with two parents reporting “A moderate amount”. Parents reported the same levels in regard to the intervention’s impact on their own well-being. Ten of 11 parents reported the intervention to be “Extremely helpful”, and 11 of 11 reported being “Very likely” to recommend the Ohana Project to another childhood cancer family. 

Open-ended responses to select questions are presented in Table 2. TCM (6/11) and social support (6/11) were most frequently mentioned as the intervention’s most valuable aspects, followed by the Facebook group prompts (4/11), health behavior education (3/11), and yoga (2/11). If parents could remove one thing about the intervention, they would reduce the frequency of the education sessions (1/11), Facebook-posting (1/11), and change the times of the education sessions (2/11). Eight of 11 parents requested removing nothing. If parents could add one thing to the intervention, they would add a child education and yoga component (4/11), more cooking classes (2/11), use a different platform than Facebook (1/11), include family counseling (1/11), include one-on-one peer support (1/11), and have a fathers-only group (1/11). Three common themes emerged from the question, “What would you tell another family about the Ohana Project?” Belongingness (8/11), represented in words like “community” and “family”; experiential peer support (8/11), represented in phrases such as “they understand what you have gone through”, and “going through a similar journey”; and education (7/11), represented in words like “guidance”, “growing”, “learn”, and “research”.

### 3.3. Exploratory Outcomes

**Figure 3:**
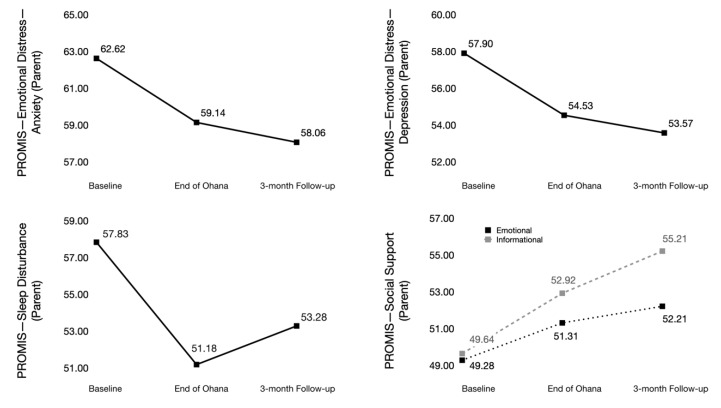
Changes in T-scores for exploratory outcomes for parents. PROMIS: the Patient-Reported Outcome System.

**Figure 4:**
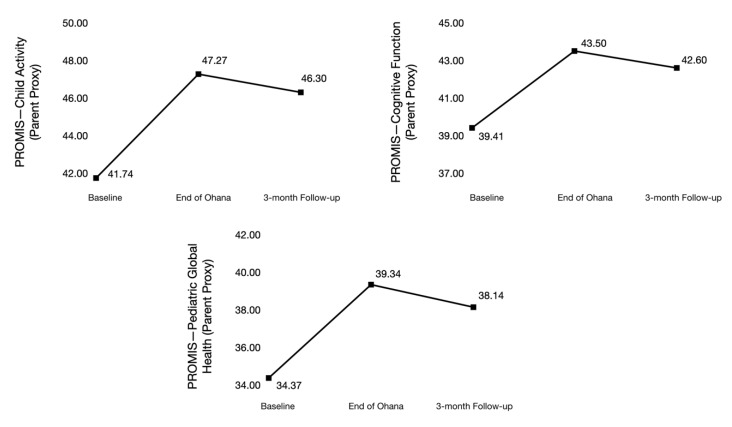
Changes in T-scores for exploratory outcomes for pediatric brain tumor patients.

At baseline, parents, on average, reported clinically relevant levels of depression and anxiety, with mean T-scores of 57.9 ± 10 and 62.2 ± 7.6, respectively. As presented in Figure 3, parent depression and anxiety T-scores decreased on average by 4.3 ± 2.4 and 4.6 ± 1.9 from T1–T3, respectively. Parent sleep disturbances decreased on average by 4.6 ± 4.3, while emotional and information support increased by 2.9 ± 1.9 and 5.6 ± 1.9, respectively, from T1-T3. As shown in Figure 4, at baseline, child physical activity was 41.7 ± 5.7 and improved by 4.7 ± 2.9 by T3. Child cognitive function and QoL T-scores were 39.4 ± 7 and 34.4 ± 12, respectively, at baseline. By T3, the child cognitive function improved by 3.2 ± 1.6 and child QoL by 3.8 ± 1.8.

## 4. Discussion

This 12-week feasibility study showed high levels of adherence and usability for its weekly group TCM and daily Facebook support group components but moderate-to-low levels of adherence and usability for its weekly parent-only education and yoga sessions. However, all components were seen by parents to be highly valuable, with every parent reporting the highest likelihood of recommending the program to other childhood cancer families. Exploratory outcomes measured at the baseline, end of the program, and the three-month follow-up suggested sustained improvements in the measures of parent emotional distress, social support, and sleep disturbances and children’s physical activity, cognitive outcomes, and QoL.

The findings suggested that a 12-week intervention that combines weekly face-to-face group interactions augmented with online Facebook group interactions is feasible, convenient, and valuable for parents of PBTPs. As a multimodal, one-arm feasibility study, no inferences can be made regarding its efficacy on exploratory psychosocial and health behavior outcomes, nor on the relative efficacy of any one component. However, from survey and qualitative data, we can infer that parents saw a high level of value in the program components for their children and for themselves. The peer support embedded in each program component was a key aspect of parents’ satisfaction with the program. The group TCM received very strong endorsements from parents, as it was seen as a unique opportunity for parents and children to connect with one another. In open-ended responses, parents requested more of these opportunities in the form of additional cooking classes (a cooking class was offered as one of the health behavior education sessions) and unstructured group gatherings. The Facebook groups were also strongly endorsed, as they were the daily conduit for sharing preventive health behavior education and behavioral prompts, as well as a platform for parents to interact with each other in between the weekly face-to-face components. The education that was shared online and in-person was referenced by parents as being valuable as a source of actionable information in the context of a disempowering health crisis. 

Since face-to-face programs require substantial resources (space, personnel, and materials) and the coordination of many diverse family schedules, it is challenging to implement them more frequently than once a week. Social media platforms represent an innovative way to supplement face-to-face programs, allowing daily communication. The Facebook component provided a high degree of privacy (“secret” groups are invite-only, cannot be seen by nonmembers, and their content cannot easily be shared outside the group); asynchronous communication accommodating varying schedules; their informational functionality facilitating the display and storage of files, photos, and video; and the flexibility of advance group posting allowing for ample planning and coordination by program designers and group moderators. In the present study, the Facebook group was not only a medium for activating theory-based social interactions, but it also served as an unstructured conduit for continuing the peer support that emerged from the weekly face-to-face interactions. 

Other studies have shown that blending face-to-face intervention components with Facebook groups is feasible, acceptable, and potentially effective at potentiating face-to-face health behavior change mechanisms [48,49,50]. Since such studies have mostly targeted weight loss in noncancer populations, the favorable results in the present study suggest that blending online and offline support may be beneficial to QoL and health behavior changes in diverse contexts. To our knowledge, interventions using Facebook groups have not targeted families of pediatric brain tumor patients specifically or childhood cancer patients more broadly. Several online [51,52] and offline [53,54,55] interventions for the parents of children affected by cancer have been conducted and have shown encouraging results for psychosocial outcomes such as depression, anxiety, post-traumatic stress symptoms, and family functioning. Parent adherence and satisfaction with the Facebook group component of this intervention suggests a cost-effective addition to these already promising models. 

This study has several strengths and limitations. Its first strength is its inclusion of a diverse group of stakeholders in its design. Between the perspectives of neurosurgery, clinical psychology, in-patient acupuncture, health behavior design, and caregiving, the study design team had a strong understanding of the latent therapeutic potential of peer support in the context of childhood cancer caregiving. Each component was designed intentionally to maximize peer interaction. The relatively small improvement in the PROMIS emotional support scale between T1, T2, and T3 may have been due to the instrument’s focus on offline significant relationships rather than gauging the unique benefits of belongingness and experiential support that peer support provides [40,45]. Another study strength is its focus on the parents of pediatric brain tumor patients. Evidence shows that, while childhood cancer patients and survivors in general suffer from poor QoL, brain tumor patients—and survivors, in particular—fare worse [3,4,5]. The effect of poor functional outcomes has a lasting effect on parent psychosocial outcomes, which may in turn feed back to contribute to poor long-term outcomes for pediatric brain tumor survivors [56]. This multimodal program is particularly promising, as it suggested potential sustained improvement in QoL outcomes for these vulnerable patients. 

As a pilot study, the small sample size and one-arm design represent obvious limitations to its generalizability, especially in regard to its exploratory outcomes. Despite recruiting a relatively diverse sample, the adherence, usability, and acceptability findings also may not be generalizable beyond Orange County, California, USA. While ethnically and socioeconomically diverse, this region has a higher average income and education than the state and the country. Childhood cancer families from other regions may find acupuncture, health behavior education, or Facebook groups to be less usable and satisfactory. Finally, parents may have been enthusiastic to join a study with free acupuncture and facilitated peer support in a community setting, which could have incentivized short-term behavior change. A larger study with an attention control group and a longer follow-up assessment is needed.

## 5. Conclusions

Peer support is an important resource for improving parent and child preventive health behaviors and QoL. Secret Facebook groups represent a low-cost, low-burden, but under-utilized peer support adjunct to intervention models for improving outcomes among parents and children affected by childhood cancer. This pilot study indicates that a multimodality peer support-based intervention that includes TCM and daily Facebook group interactions is feasible and acceptable to parents of pediatric brain tumor patients. Further research on interventions for this population that include TCM and Facebook group-based peer support is warranted.

## Figures and Tables

**Table 1:** Parent demographics and children’s clinical characteristics.

Variable	Parents (*n* = 12)	Children (*n* = 10)
**Age, years: mean (SD)**	38.3 (7.8)	7.8 (3.5)
**Gender (%)**		
**Female**	10 (83%)	6 (60%)
**Male**	2 (17%)	4 (40%)
**Ethnicity (%)**		
**Non-Hispanic white**	5 (42%)	
**Hispanic or Latino**	7 (58%)	
**Diagnosis (%)**		
**Atypical Teratoid Rhabdoid**		1 (10%)
**Ependymoma**		1 (10%)
**Low-grade glioma**		4 (40%)
**Pineoblastoma**		3 (30%)
**Treatments Received (%)**		
**Surgery only**		3 (30%)
**Surgery + chemotherapy**		1 (10%)
**Surgery + chemo + radiation**		6 (10%)
**Treatment status (%)**		
**Still in treatment**		5 (50%)
**Less than 2 years off treatment**		1 (10%)
**More than 2 years off treatment**		4 (40%)
**Marriage Status (%)**		
**Married**	9 (75%)	
**Separated**	0 (0%)	
**Divorced**	0 (0%)	
**Never married**	3 (25%)	
**Education**		
**Some College or below**	7 (58%)	
**College degree or above**	5 (42%)	

**Table 2:** Complete responses of parents to open-ended acceptability questions at the end of the intervention (T2). TCM: Traditional Chinese medicine.

ID	What was the most valuable part of the Ohana Project for you and your child?
01	TCM, educational presentations, and the Facebook interaction. I loved seeing what other people wrote and recipes/pics and the OHANA things to strive for everyday. All of it was so valuable.
02	I really liked the Facebook post of what people were doing and the encouragement. The first four weeks were my favorite. We had small, manageable goals. Posting was my favorite. Sometimes yoga was hard for me on Sundays but it was always good.
03	Learning in the Facebook group more about how to eat properly. Acupuncture, meditation, importance of sleep habits and vigorous exercise.
04	Connecting with other parents and kids on a similar journey. We don’t feel lost and alone in this anymore.
05	Acupuncture
06	The [acupuncture] appointments were the best. Parent yoga was a close second.
07	Everything was great but definitely having the support of other parents going through the same thing was super valuable. Our time receiving acupuncture in a group setting was priceless, therapeutic and comforting.
08	For me I would say it was the community aspect. I felt grateful to be around other families going through the same journey as my child and I. The knowledge and support I received from [the Facebook group] are irreplaceable.
09	Acupuncture!
10	The most valuable part was being part of a community that understood what we were going through. We all felt appreciated.
11	The connections with other families. It was really just a great group of families.
	**What would you tell another cancer family about the Ohana Project?**
01	The Ohana project was so helpful to be around other parents and families that are on the same level as you. They understand what you have gone through or are going through. Acupuncture has become a regular routine for us and our daughter feels she needs it to function in the week.
02	It is really good for new parents that have just been given the diagnosis. It creates an automatic community for them. It’s also good for those who have been in the fight longer because it’s very easy to get off course.
03	It provides a community of like-minded people experiencing similar challenges. It provides access to peer-reviewed, proven scientific data and the experts that support such data on health & well-being.
04	As parents we would do ANYTHING to take our child’s pain or discomfort or sadness away. The Ohana Project gives parents tangible things to do to help our children thrive during treatment and beyond. The Ohana Project connects parents and children going through a similar journey and provides them with evidence-based research and classes, acupuncture, and social support through those new family connections. It is such an impactful and life changing program that I feel provides the missing link in healthcare.
05	Great information, guidance and support
06	It’s refreshing to be surrounded by others who are going through the exact same thing. It’s a judgement free zone.
07	It was our saving grace! Being recently diagnosed, it helped bring us out of our DARK place.
08	Trust that it is a process. Do not be so tough on yourself, it’s not a race better yet a marathon to the best version of health, community, and thriving during and after treatment.
09	This was a growing period for our family and many changes were made for the positive. Having support of other like-minded people was life changing for us!
10	Every family that has a child battling cancer should experience the Ohana Project. The small community support is so important.
11	The Ohana project is exactly like the name “a family” of parents and children going through the same journey as you are. A place where children can meet and play with kids just like them, going through the same experiences they are. Where parents can share and learn from one another without judgment.
